# Characterization of measurement errors using structure‐from‐motion and photogrammetry to measure marine habitat structural complexity

**DOI:** 10.1002/ece3.3127

**Published:** 2017-06-15

**Authors:** Mitch Bryson, Renata Ferrari, Will Figueira, Oscar Pizarro, Josh Madin, Stefan Williams, Maria Byrne

**Affiliations:** ^1^ Australian Centre for Field Robotics University of Sydney Sydney NSW Australia; ^2^ School of Biological Sciences University of Sydney Sydney NSW Australia; ^3^ Department of Biological Sciences Macquarie University North Ryde NSW Australia; ^4^ Schools of Medical and Biological Sciences University of Sydney Sydney NSW Australia

**Keywords:** 3D habitat mapping, coral ecology, photogrammetry, structural complexity, structure‐from‐motion

## Abstract

Habitat structural complexity is one of the most important factors in determining the makeup of biological communities. Recent advances in structure‐from‐motion and photogrammetry have resulted in a proliferation of 3D digital representations of habitats from which structural complexity can be measured. Little attention has been paid to quantifying the measurement errors associated with these techniques, including the variability of results under different surveying and environmental conditions. Such errors have the potential to confound studies that compare habitat complexity over space and time. This study evaluated the accuracy, precision, and bias in measurements of marine habitat structural complexity derived from structure‐from‐motion and photogrammetric measurements using repeated surveys of artificial reefs (with known structure) as well as natural coral reefs. We quantified measurement errors as a function of survey image coverage, actual surface rugosity, and the morphological community composition of the habitat‐forming organisms (reef corals). Our results indicated that measurements could be biased by up to 7.5% of the total observed ranges of structural complexity based on the environmental conditions present during any particular survey. Positive relationships were found between measurement errors and actual complexity, and the strength of these relationships was increased when coral morphology and abundance were also used as predictors. The numerous advantages of structure‐from‐motion and photogrammetry techniques for quantifying and investigating marine habitats will mean that they are likely to replace traditional measurement techniques (e.g., chain‐and‐tape). To this end, our results have important implications for data collection and the interpretation of measurements when examining changes in habitat complexity using structure‐from‐motion and photogrammetry.

## 1 INTRODUCTION

Habitat structural complexity is one of the most important factors in structuring biological communities. In marine environments, there exists a wealth of studies linking increases in habitat complexity to increases in abundance and diversity of both benthic and mobile organisms (Graham & Nash, 2013; Gratwicke & Speight, 2005; Harborne, Mumby, Kennedy, & Ferrari, 2011; Kovalenko, Thomaz, & Warfe, 2012; Luckhurst & Luckhurst, 1978; Meager, Schlacher, & Green, 2011; Rees, Jordan, Price, Coleman, & Davis, 2014). Structurally complex habitats may promote biodiversity and abundance of biota through increasing habitat niches and/or increased habitat availability (Johnson, Frost, Mosley, Roberts, & Hawkins, 2003; Willis, Winemiller, & Lopez‐Fernandez, 2005). In coral reef ecosystems, structural complexity has been identified as one of the most important attributes in determining reef resilience (Graham, Jennings, MacNeil, Mouillot, & Wilson, 2015) and whether a reef community returns to a coral‐dominated or shifts to an algal‐dominated state following disturbance.

In marine benthic habitats, structural complexity is measured in a variety of ways including both qualitative (e.g., visual assessment Lara & Gonzalez, 1998; Wilson, Graham, & Polunin, 2007; Graham et al., 2015) and quantitative (e.g., chain‐and‐tape Luckhurst & Luckhurst, 1978 and profile/level gauge McCormick, 1994) techniques. Recently there has been a proliferation in studies using photogrammetry and structure‐from‐motion techniques for measuring structural complexity in underwater habitats (e.g., Burns, Delparte, Gates, & Takabayashi, 2015; Burns et al., 2016; Ferrari, Bryson, et al., 2016; Figueira et al., 2015; Friedman, Pizarro, Williams, & Johnson‐Roberson, 2012; Leon, Roelfsema, Saunders, & Phinn, 2015). These techniques use a series of overlapping images, taken from multiple perspectives to reconstruct the three‐dimensional (3D) structure of the seafloor and habitat‐forming organisms at high resolution and accuracy, from which structural complexity measurements can then be derived. Structural complexity is commonly represented by a rugosity index that is the ratio of habitat surface area to the total areal extent of the habitat (Friedman et al., 2012). This measure of complexity is an area‐based equivalent to the linear transect measurements available using a traditional chain‐and‐tape or profile techniques and provides a more specific representation of habitat complexity present (Friedman et al., 2012). Unlike chain‐and‐tape methods, the generation of 3D models allows for the spatial resolution of complexity measurements (equivalent to the link size in chain‐and‐tape methods) to be easily controlled and varied (Ferrari, Bryson, et al., 2016), a feature important for comparing complexity measurements (Knudby & LeDrew, 2007) but frequently ignored or unreported (Graham & Nash, 2013). Furthermore, photogrammetric methods allow for habitats to be measured over larger areas than traditional techniques (Leon et al., 2015) and for measurements to be made in a noninvasive fashion (Bridge et al., 2014).

The underwater environment provides many challenges to photogrammetry not encountered in above‐water settings; large variations in ambient light and water clarity affect image quality and control over the precise position and orientation from which photographs are acquired is difficult. Although the use of photogrammetric techniques in underwater environments is not new (see, e.g., Done, 1981), its application has been vastly simplified in recent years with the availability of software (such as Agisoft PhotoScan [http://www.agisoft.com] and Visual SFM [http://ccwu.me/vsfm]) that can process data without the need for ordered images, detailed ground control or prior camera calibration that previous photogrammetry methods have relied on. Although these techniques can be employed with great success, they are inherently complex, relying on a plethora of factors including image quality, resolution, image textural properties, camera lens distortions and artifacts, surface brightness, shape, and roughness. What is lacking in recent studies using these techniques is a clear understanding of how these factors influence and induce errors in measurements of structural complexity. In the context of measuring changes in habitat complexity, these factors have the potential to confound studies where factors affecting measurement error vary across space and time. Characterization of these errors is crucial for proper inference of changes in structural complexity over time with respect to changes in other covariates such as metrics of biological assemblage structure that change across disturbance events.

The purpose of this study is to quantify the accuracy, precision and potential bias of ecologically relevant mesoscale (tens to hundreds of meters) structural complexity measurements of marine benthic habitats determined from structure‐from‐motion photogrammetry. On coral reefs, existing ecologically focused evaluations into the accuracy of underwater photogrammetry have focused primarily at the scale of individual coral colonies (Bythell, Pan, & Lee, 2001; Courtney, Fisher, Raimondo, Oliver, & Davis, 2007; Lavy et al., 2015). These studies use survey techniques that image a single, small region of space around the colony, where occluding objects are not present in the scene, and from multiple perspectives and ranges to the target. Lavy et al., 2015 found photogrammetric techniques resulted in accuracies of single‐colony surface area ranging from 2% to 18% of the total surface area, depending on colony shape. Courtney et al., 2007 found errors less than 12% in computed coral volume. The resulting quality of 3D models is not indicative of the performance of these methods when applied to more extensive regions of the benthos, where habitat patchiness results in a complex, interwoven network of colonies, and survey techniques cannot be tailored to each individual colony. In recent studies (Ferrari, Bryson, et al., 2016; Ferrari, McKinnon, et al., 2016; Figueira et al., 2015) , measurement precision was considered for both colony scale and patch scale (19 × 6 m regions of temperate and tropical reefs), but these works did not study the driving factors behind the precision of the approach.

Our study addresses the following questions:

1. How accurate are complexity measurements derived from underwater structure‐from‐motion and do measurements underestimate or overestimate complexity?
2. Do changes in environmental conditions (lighting and water quality) between surveys of the same area across different times result in variation in surface rugosity (SR) measurements?
3. How do measurement errors change as a function of:
  - Survey trajectory, image density, and image coverage changes?
  - Actual SR?
  - The differing community composition and abundance of habitat‐forming organisms (i.e., coral) with differing morphologies?

Using coral reef habitats as a case study, we use repeated measurements over an artificial reef scene (with known 3D structure) and shallow water coral reefs on the Great Barrier Reef, Australia, to quantify both accuracy (difference from the “true” SR) and precision (variability in measured structural complexity) of photogrammetric measurement techniques. Measurement errors are examined in the context of different survey techniques, changing ambient lighting conditions, and differences in water clarity. We develop models of complexity measurement errors as a function of varying image coverage pattern, actual SR, and changes in dominant coral morphology. In the context of using structural complexity as a measure of reef condition, these models have the potential to inform decisions about how data should be collected and processed. Where repeated measurements are made, such characterization is crucial for understanding what level of structural complexity change might be detectable, for example, given prior knowledge of the complexity and habitat‐forming communities present.

## 2 MATERIALS AND METHODS

### 2.1 Study sites

**Figure 1:**
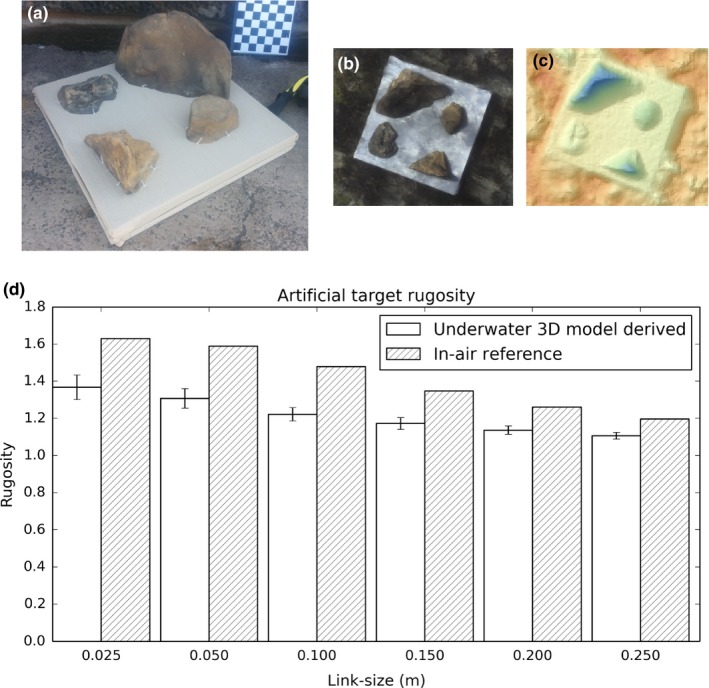
(a) Artificial reef used to evaluate reconstruction accuracy and repeatability, (b) underwater photomosaic of artificial reef, (c) corresponding digital surface model colored by relief height. (d) Comparison of rugosity measurements derived from 3D models reconstructed using diver‐rig underwater stereo imagery and rugosity measurements from an in‐air reference model, reconstructed using in‐air high‐resolution images

Initial experiments using a 1.1‐by‐1.1 m artificial reef (see Figure 1) were performed in an ocean swimming pool in Sydney, Australia (33.9684°S, 151.2546°E), and experiments over coral reefs were performed at Lizard Reef (14.6680°S, 145.4617°E) and Heron Reef (23.4423°S, 151.9148°E), Great Barrier Reef, Australia. Image data at Lizard Reef were collected at two survey sites during October 2014 (Horseshoe Reef) and December 2015 (Turtle Beach) located on reef flats within the lagoon in depths of 1.5–2.0 m. Benthic communities at these sites were dominated by massive (*Favia* ssp. and *Porites* spp.) and Fine‐Branching corals (*Acropora* spp.). Image data were collected at two survey sites at Heron Reef during January 2016 located along the outer slope of the lagoon (Blue Pools and Harry's Bommie) in depths of 2.5–5.0 m. The Blue Pools site was dominated by plating coral morphotypes (*Montipora* spp.), whereas the Harry's Bommie site was dominated by a combination of coarse and Fine‐Branching (*Acropora* ssp.) and plating coral.

### 2.2 Equipment

**Figure 2:**
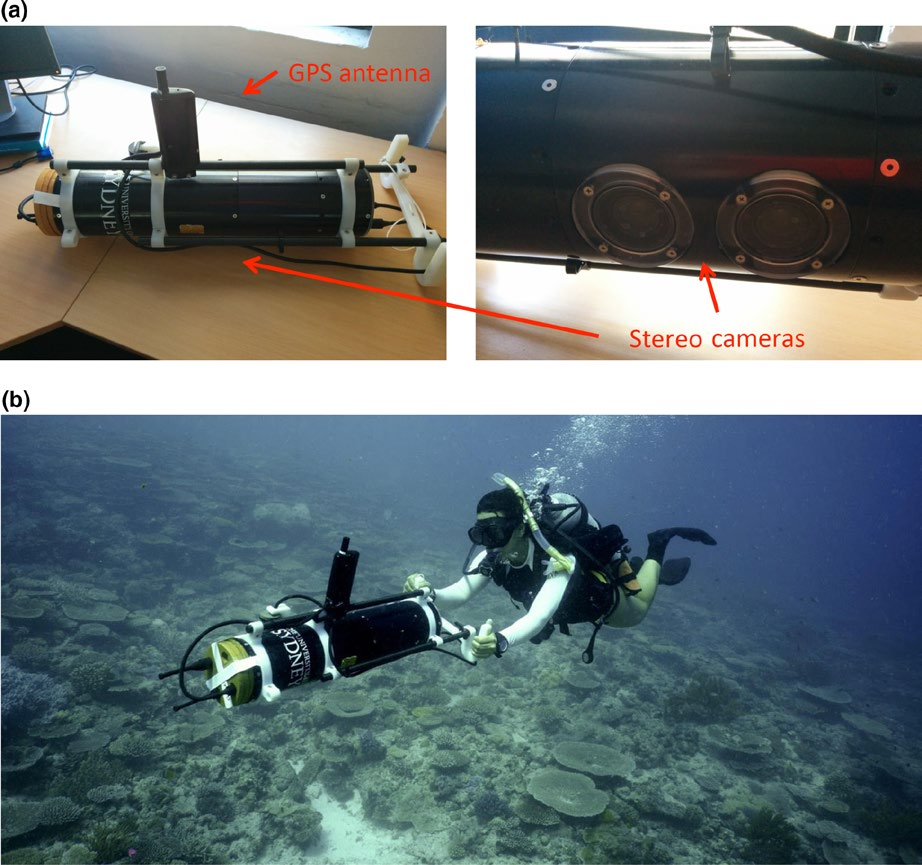
(a) Diver‐operated stereo camera rig (diver‐rig) containing stereo camera pair, depth pressure sensor, tilt sensors, magnetic compass and GPS receiver, (b) diver‐rig in operation during surveys (photograph by Chris Roelfsema)

A diver‐operated stereo camera rig (Figure 2, referred herein as the “diver‐rig”) was used to collect imagery over survey sites on the seafloor to build image‐derived 3D topographic surface models. The diver‐rig carried a downwards‐looking stereo camera pair utilizing one color and one monochrome Prosilica GC1380 12 bit camera, each with a resolution of 1,360 × 1,024 pixels and an imaging field of view of 42^°^ × 34^°^ in water, providing a ground sampling distance of ~1 mm per pixel over a 1.5 × 1.2 m footprint at 2 m from the substrate. The diver‐rig also carried a pressure‐depth sensor, tilt sensors, a magnetic compass, and Global Positioning System (GPS) receiver (data available when operating at the surface). Data from these sensors were used to assist in the image‐based structure‐from‐motion and 3D surface reconstruction process using custom‐designed software (Bryson, Johnson‐Roberson, Pizarro, & Williams, 2013; Johnson‐Roberson, Pizarro, Williams, & Mahon, 2010).

### 2.3 Data collection and survey techniques

Imaging surveys using the diver‐rig were conducted at each site and repeated over 4 days at each location. During each survey, overlapping images of the site were captured at 2 m from the substrate with a ground spacing distance between images of approximately 50 cm.

Two survey techniques were used to guide the trajectory of the diver‐rig over the terrain, depending on the reef environment type. When operating over reef flats (Horseshoe Reef and Turtle Beach), the “Reef Record” (Pizarro, Friedman, Bryson, Williams, & Madin, 2017) data collection protocol was used. A 6‐m line was connected to and coiled around a 16‐cm‐diameter drum mounted to a survey pole affixed to the center of the survey region, while the other end of the line was then connected to the diver‐rig. The diver‐rig was then pushed through the water in an outwards spiral trajectory keeping the line taut resulting in a consistent track space of 50 cm at each subsequent turn of the spiral. The resulting imagery provided contiguous coverage over a circular plot of the terrain 12 m in diameter, covering approximately 100 m^2^ of the seafloor.

When operating over sloping terrain (Blue pools and Harry's Bommie), a “Mow‐the‐lawn” trajectory was followed by the diver, in which the diver guided themselves according to visual markers placed in the survey area prior to imaging. A series of approximately 14–17 visual markers (pink flagging tape) were placed on the seafloor along a distance of 30 m following the depth contour. The diver pushed the diver‐rig through the water in straight lines back and forth between the two markers while adjusting the cross‐track horizontal position of the trajectory line 50 cm along the direction of the slope at each subsequent pass, such that a 30 × 6 m (approximately 180 m^2^) area was covered.

### 2.4 Structure‐from‐motion postprocessing

**Figure 3:**
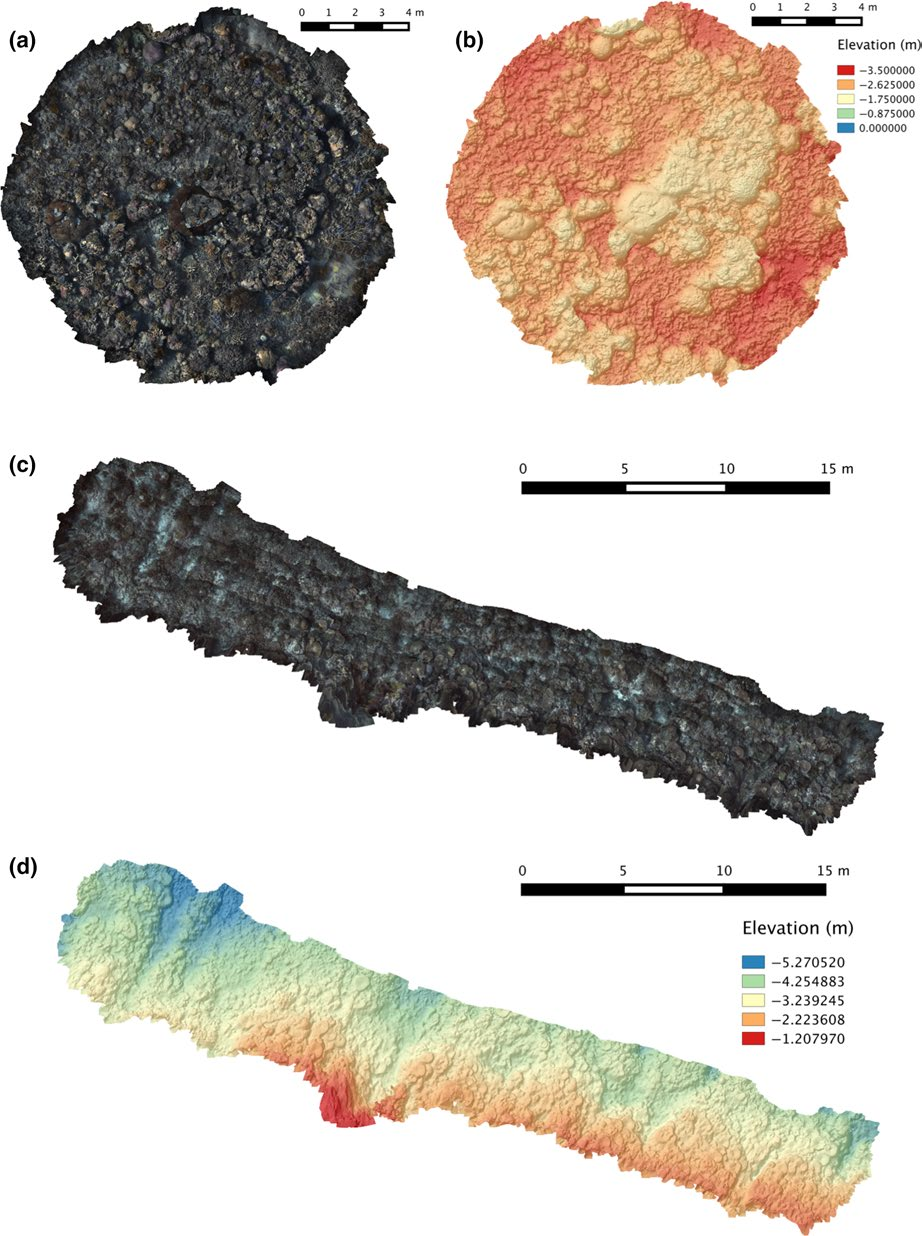
(a) Orthographic imagery mosaic and (b) topographic surface model at Horseshoe Reef survey site, Lizard Island using “Reef Record” survey method. (c) Orthographic imagery mosaic and (d) topographic surface model at Blue Pools survey site, Heron Island using “Mow‐the‐Lawn” survey method

For each survey of each site, collected images and other diver‐rig sensor data were used to reconstruct photomosaics and 3D surface models of the seafloor (Figure 3). Estimation of the position and orientations (poses) of the stereo pairs was performed using a feature‐based stereo bundle adjustment algorithm (Johnson‐Roberson et al., 2016). A similar procedure is used by commercially available structure‐from‐motion/photogrammetry software such as Agisoft PhotoScan (http://www.agisoft.com/), the main difference in our approach being the use of a precalibrated stereo camera system. Camera calibration parameters were ascertained using the OpenCV Camera Calibration Toolbox (http://docs.opencv.org/2.4/modules/calib3d/doc/camera_calibration_and_3d_reconstruction.html), using images of a black and white checkerboard target taken prior to fieldwork activities. Once camera poses had been recovered, a terrain reconstruction algorithm (Johnson‐Roberson et al., 2010) was used to produce a topographic surface model of the imaged area.

### 2.5 Calculation of surface rugosity

Topographic surface models were used to derive multiscale measures of structural complexity using SR, the ratio of total surface area of the model divided by the projected surface area of the sampled region onto a plane. For each survey site, a plane of best fit to the overall site surface was calculated using a least squares fit and used as the axis of projection for SR measurements, to decouple measurements of SR from the overall slope of the environment. Each topographic surface model was divided into nonoverlapping virtual quadrats (2 × 2 m), and one value of rugosity was extracted from each virtual quadrat, for each survey using Equation (1):

(1) $$\text{SR}=\frac{\sum_{N}^{i=1}a_{i}}{\sum_{N}^{i=1}a_{\mathrm{proj},i}}$$

where *a*_*i*_ is the actual area of a surface face element *i*,* i *=* *1 to *N* (all faces in a given virtual quadrat), and *a*_proj,*i*_ is the orthographically projected area of face *i*, corresponding to the coordinate system based on the plane of best fit. SR measurements were firstly computed using the full resolution topographic surface models (2.5 cm resolution) and subsequently computed on several down‐sampled versions of each mesh at increments of 5 cm up to 25 cm resolution to generate SR measurements across at a range of link‐size resolutions.

### 2.6 Surface rugosity measurement repeatability and error estimation

To assess the accuracy and precision of structural complexity measurements derived from the resulting topographic surface models, the surveys were repeated multiple times at each site over varying temporal scales. Once surface models from each survey of a given site had been produced, they were spatially registered into a common reference frame using a rigid six‐degree‐of‐freedom transformation (Bryson et al., 2013) with a residual accuracy of ±5 cm. The precisely registered surface models allowed for measurements to be compared within the 2 × 2 m virtual quadrats across multiple surveys.

Given the logistical challenges of imaging a natural reef in air, we used an artificial reef to assess accuracy of the SR measurements (see Figure 1). At the Sydney ocean swimming pool, the artificial reef was placed into the water and surveyed eight times on the same day using a Mow‐the‐lawn survey pattern, from which eight separate reconstructions of the same area were built. The accuracy of the SR measurements was ascertained by examining the difference in SR between topographic surface model‐derived measurements and those derived from a reference model of the surface (artificial reef), constructed using high‐resolution in‐air photogrammetry. The reference surface model was reconstructed using four hundred photographs (each 16 megapixel resolution) taken from a variety of orientations to reconstruct a 5 mm per spatial point model with an accuracy of ±5 mm, based on feature residual analysis. Reference measurements of SR were obtained from the model at several link‐size resolutions (from 2.5 to 25 cm) and compared to the corresponding measurements derived from the underwater surface models, ensuring resolutions were appropriately matched for each comparison.

The precision (repeatability) of SR measurements was assessed using the repeated surveys over natural coral reefs by comparing the distribution of measurements across surveys to the average SR. At each site, four surveys were performed on four different days and at three of the sites (Blue Pools, Harry's Bommie and Turtle Beach) surveys were also performed four times back‐to‐back during a 1‐hr period in a single day (single‐day surveys not performed at Horseshoe site owing to logistical restrictions at the study site). Data from each survey were used to reconstruct separate topographic surface models, one for each survey. An average SR measurement across all available surveys was computed for each 2 × 2 m virtual quadrat. SR measurements errors for each quadrat were then estimated by taking the standard deviation of differences from this average:

(2) $$\sigma_{\mathrm{SR},j}=\sqrt{\frac{1}{N_{\mathrm{survey}}}\;\sum_{N_{\mathrm{survey}}}^{i=1}{\left(\delta {\text{SR}}_{j,i}\right)}^{2}}$$

(3) $$\delta {\text{SR}}_{j,i}={\text{SR}}_{j,i}-\mu_{\mathrm{SR},j}$$

where σ_SR*,j*_ is the SR measurement standard error of quadrat *j*,* N*_survey_ is the number of surveys over quadrat *j*, SR_*j,i*_ is the SR measurement for quadrat j from survey *i*,* i *=* *1 to *N*_survey_, and μ_SR*,j*_ is the average SR measurement for quadrat *j*:

(4) $$\mu_{\mathrm{SR},j}=\frac{1}{N_{\mathrm{survey}}}\;\sum_{N_{\mathrm{survey}}}^{i=1}{\text{SR}}_{j,i}$$

Estimates of the per‐survey bias (*b*_*i*_) for each site were generated (one value for each survey of each site) by averaging these differences across all quadrats at a given site:

(5) $$b_{i}=\frac{1}{N_{\mathrm{quad}}}\sum_{N_{\mathrm{quad}}}^{j=1}\delta {\text{SR}}_{j,i}$$

Measurements for each quadrat were also grouped according to surveys taken back‐to‐back on the same day (*N* = 4) and surveys taken over multiple days (*N* = 4) to generate two separate estimates of measurement error and bias (single day and multiday), corresponding to similar and different environmental conditions (Table 1).

**Table 1:** Summary of data collection and processing details

Site	Artificial Reef	Horseshoe Reef	Turtle Beach	Blue Pools	Harry's Bommie
Site topography/survey method	Flat, Mow‐the‐lawn	Flat, Reef Record	Flat, Reef Record	Slope, Mow‐the‐lawn	Slope, Mow‐the‐lawn
Survey date	07/2016	10/2014	12/2015	01/2016	01/2016
Single‐day surveys	8	0	4	4	4
Multiday surveys	0	4	4	4	4
Average survey time	71 s	20 min	13 min	13 min	12 min
Average stereo pairs	142	2,426	1,605	1,587	1,425
No. virtual quadrats	N/A	46	32	48	65

### 2.7 Annotation of coral morphotypes

Imagery mosaics were digitally annotated by proportion of the benthic classes: “Sand,” “Abiotic/Mixed Hard Bottom,” and coral morphotypes “Massives,” “Plating,” “Fine‐Branching,” and “Coarse Branching” using GIS. The abundance of each class in each virtual quadrat was calculated and quadrats were assigned into categories according to the dominant coverage type (highest area proportional to other labeled classes in the quadrat), when the dominant coverage class abundance was >25%. When the greatest abundance in a quadrat was <25%, the quadrat was assigned as “Mixed.”

### 2.8 Image coverage metrics

Image coverage metrics for each virtual quadrat were computed by creating an additional spatial layer on top of the topographic surface models that measured the number of images that observed each spatial point in the quadrat, reproduced at a resolution of 0.5 cm per pixel. Two metrics were calculated: the first which measured the average coverage of a quadrat (average number of images observing each spatial point in the quadrat) and the second which measured the coverage variation (standard deviation of the number of images observing each spatial point in the quadrat).

### 2.9 Statistical analyses

Accuracy of the in‐water topographic surface models was tested using two‐tailed *t*‐tests comparing the average in‐water SR measurements to the SR measurements derived from the in‐air reference surface model (Question 1).

To examine the effects of varying environmental conditions and varying survey trajectory (Question 2), distributions of the quadrat SR measurements per survey and standard error (precision) were compared for surveys across a single day versus surveys conducted across multiple days. Two analyses were performed. For the first analysis, paired sample *t*‐tests were used to examine whether the distributions of quadrat SR standard error (σ_SR*,j*_) varied when standard error was computed using only single‐day surveys or only surveys over multiple days. For the second analysis, one‐way analysis of variance (ANOVA) was used to test the hypotheses that the mean of SR measurements differences (δSR_*j,i*_) for each survey *i* were statistically different. Separate tests were performed for each site and for differences among surveys performed on the same day and surveys performed across multiple/different days. Data exploration techniques were used to validate assumptions of normality and homogeneity of variance when performing analyses (Zuur, Ieno, & Elphick, 2010).

To examine the relationship between errors in measured SR and the way in which images were collected from the target site (Question 3a), correlation coefficients and ordinary least squares (OLS) fits for SR measurement standard error versus the two image coverage metrics were computed.

To examine the effect of the underlying surface on the SR measurement error and to investigate whether higher complexity surfaces yielded larger measurement errors (Question 3b), OLS was used to estimate a linear model of SR measurement error as a function of average rugosity, using quadrat‐scale data from all of the four surveys sites. In order to additionally examine the effect of the dominant coral morphotype on rugosity errors (Question 3c), this OLS model was extended to include dominant coral morphotype as a categorical variable based on the classes “Massives,” “Plating,” “Fine‐Branching,” and “Coarse Branching” using a simple contrast coding scheme with the class “Mixed” as the base reference category. For all OLS modeling, residual plots were used to verify that model assumptions were not violated. All statistical tests (OLS and ANOVA) were performed using the StatsModels python package (http://statsmodels.sourceforge.net/devel/index.html).

## 3 RESULTS

### 3.1 Artificial reef

Measurements of SR from in‐water topographic models consistently underestimated the reference model SR computed using in‐air photographs (Figure 1). T‐test results at each link size indicated that differences were statistically significant (Table 2). SR measurements were between 8% and 15% lower for in‐water reconstructions when compared to the reference reconstruction, but highly repeatable with a standard deviation of 0.065 (4.9% of the true rugosity) for the 2.5 cm link‐size resolution. Underestimation was likely due to a combination of factors including loss of contrast when imaging through the water and that the survey techniques were not able to resolve the complexity contributed by overhanging surfaces, which were not imaged during the survey techniques employed in this study, which observed surfaces from above.

**Table 2:** Results of two‐tailed Student's *t*‐tests comparing rugosity derived using underwater imagery against rugosity from an in‐air reference 3D model of an artificial reef

Link size	Reference mean	Measured mean	Measured standard deviation	*t* value (*N* = 8)	*p* value
2.5 cm	1.630	1.367	0.065	−10.52402	<.00001
5 cm	1.590	1.307	0.053	−14.21824	<.00001
10 cm	1.478	1.222	0.037	−18.51321	<.00001
15 cm	1.348	1.173	0.032	−14.64828	<.00001
20 cm	1.260	1.136	0.023	−14.27399	<.00001
25 cm	1.197	1.106	0.018	−13.33120	<.00001

### 3.2 Coral reef topographic reconstructions

**Figure 4:**
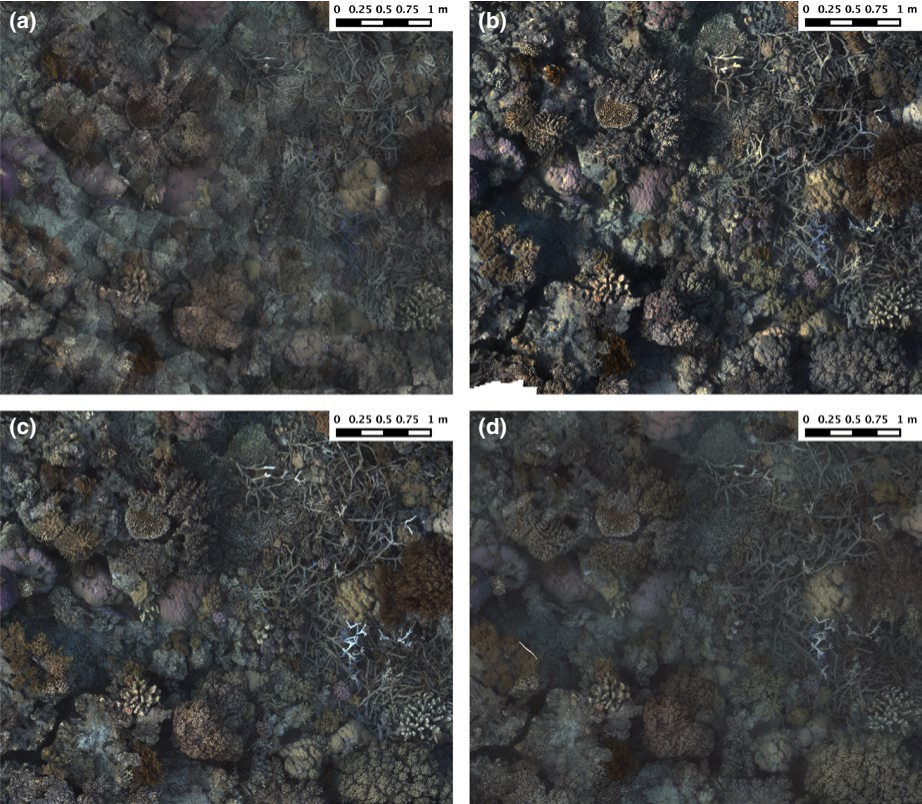
Imagery mosaics from multiday surveys at Horseshoe Reef, Lizard Island (a) Day 1, (b) Day 2, (c) Day 3, (d) Day 4

**Figure 5:**
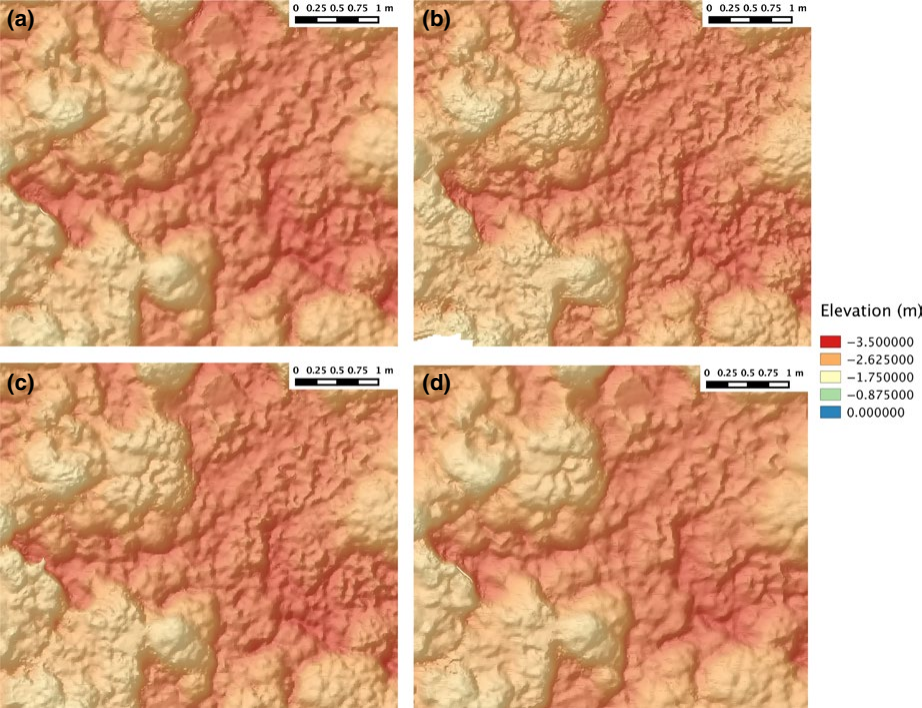
Surface models from multiday surveys at Horseshoe Reef, Lizard Island (a) Day 1, (b) Day 2, (c) Day 3, (d) Day 4

Data collection time for field surveys varied from 12 to 20 min, and each reconstruction was produced using approximately 1,400–2,400 stereo images pairs (Table 1). Variations in the ambient lighting (sunny vs. cloudy conditions and time of day) and water clarity over the multiple surveys resulted in variations in the shadow direction, color, and contrast observable in reconstructed photomosaics (Figures 3 and 4). The corresponding topographic surface models exhibited subtle variations in how well fine‐scale features were resolved (Figure 5).

### 3.3 Coral reef structural complexity measurements

**Figure 6:**
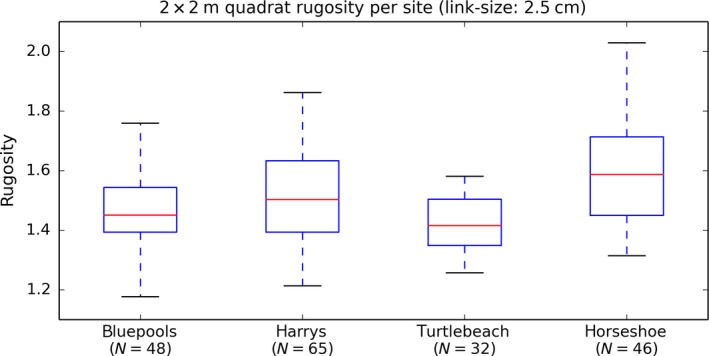
Distribution of average rugosity for 2 × 2 m virtual quadrats (μ_SR_) at each of the four reefs surveyed in this study (resolution 2.5 cm)

**Figure 7:**
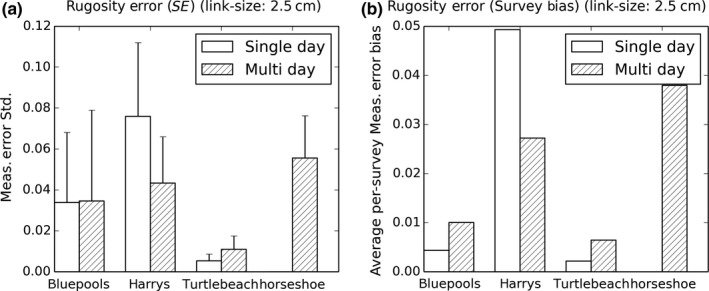
2 × 2 m quadrat rugosity measurement errors at each site: (a) 2 × 2 m quadrat standard deviation from quadrat average σ_SR_, averaged for all quadrats at each site. (b) Root mean square (RMS) of average 2 × 2 m quadrat rugosity difference from quadrat average per survey (*b*), a measure of the per‐survey bias induced by conditions for a particular survey

Habitat complexity was observed to be highly heterogeneous across the spatial range of each of our sites (12–30 m), owing to the heterogeneous benthic composition of different coral morphotypes, highlighting the importance of mesoscale sampling (Figure 6). The site‐averaged quadrat‐scale measurement standard error σ_SR_ and bias *b* varied significantly between sites and when examining single‐day versus multiday surveys at the same site (Figure 7, Table 3). Harry's Bommie had the largest standard error (11.68% of observed rugosity range) and largest error bias (7.59% of observed rugosity range) for measurements taken across either a single day or multiple days. Results of paired *t*‐tests between single‐day versus multiple‐day SR measurement standard error showed that single‐day measurement errors were significantly larger than multiple‐day errors at the Harry's Bommie site (*t*(65) = 7.35946, *p* < .00001), but that multiple‐day errors were significantly larger than single‐day errors at Turtle Beach (*t*(32) = 4.72941, *p* < .0001). No significant difference in errors between single‐day versus multiple‐day surveys was observed at the Blue pools site (*t*(48) = −0.27341, *p* = .78574).

**Table 3:** Rugosity measurement errors at each site as a percentage of observed range of rugosity values at each site

Blue pools		Harry's Bommie		Turtle Beach		Horseshoe Reef	
Single day	Multiday	Single day	Multiday	Single day	Multiday	Single day	Multiday
Rugosity standard error							
5.63%	5.76%	11.68%	6.66%	1.64%	3.43%	NA	7.82%
Rugosity per‐survey bias							
0.72%	1.67%	7.59%	4.19%	0.67%	2.01%	NA	5.34%

**Figure 8:**
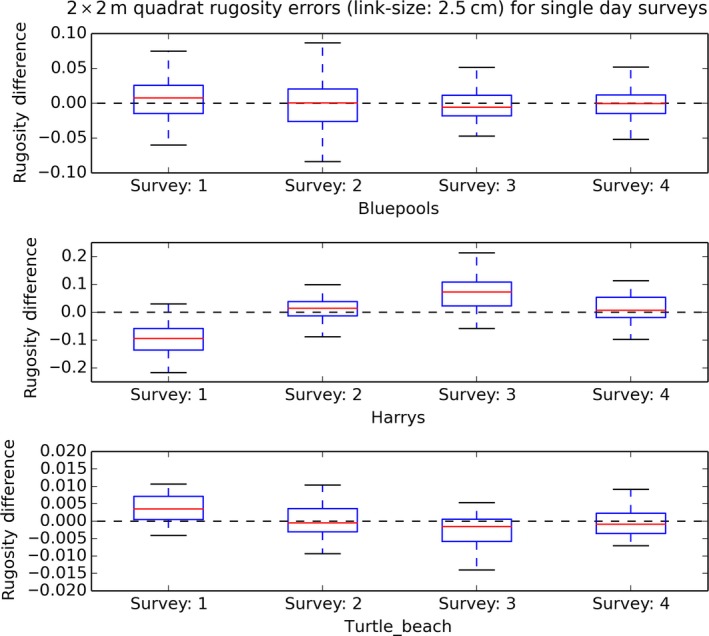
Boxplot distributions of 2 × 2 m quadrat rugosity measurement differences δSR_*j,i*_ per survey (*i *=* *1 to 4) from quadrat average across all surveys, for surveys performed on the same day. The distribution of differences from average for a given survey may be greater or less than zero, indicating that quadrats are being measured consistently higher or lower for the conditions in which the survey was performed

**Figure 9:**
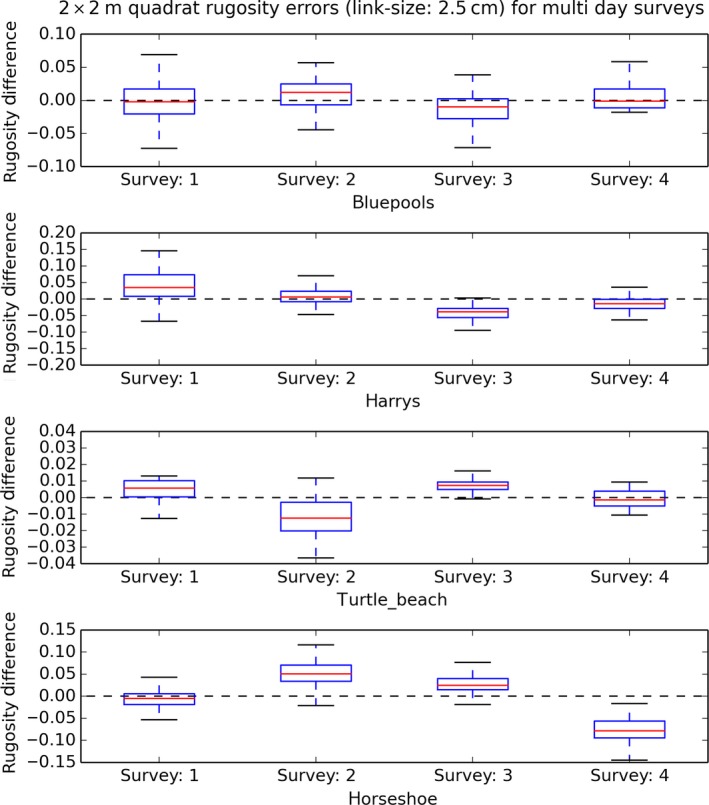
Boxplot distributions of 2 × 2 m quadrat rugosity measurement differences δSR_*j,i*_ per survey (*i *=* *1 to 4) from quadrat average across all surveys, for surveys across multiple days. The distribution of differences from average for a given survey may be greater or less than zero, indicating that quadrats are being measured consistently higher or lower for the conditions in which the survey was performed

Single‐day survey bias was smaller than multiday bias at Turtle Beach and Blue pools, but the inverse was true at Harry's Bommie. Quadrat‐scale SR measurements taken across a single survey at a time had statistically significant per‐survey biases (were consistently higher or lower across the whole survey compared to the average of other surveys) for three of the four study sites (Figures 8 and 9) when considered over both single‐day surveys and surveys over multiple days. For surveys performed on the same day at the same site (Figure 8), the mean quadrat‐scale SR measurements did not vary significantly between surveys at the Blue pools site (*F*(3,191) = 0.687804, *p* = .5605), but did vary significantly between surveys at both the Harry's Bommie site (*F*(3,259) = 99.969, *p* < .00001) and the Turtle Beach site (*F*(3,127) = 9.6489, *p* < .00001). For surveys performed across different days at the same site (Figure 9), quadrat‐scale rugosity measurements again did not vary significantly between surveys at the Blue pools site (*F*(3,191) = 2.55718, *p* = .0565), but did vary significantly between surveys at the Harry's Bommie site (*F*(3,259) = 57.1376, *p* < .00001), Turtle Beach site (*F*(3,127) = 22.1443, *p* < .00001), and Horseshoe Reef site (*F*(3,183) = 94.6408, *p* < .00001).

### 3.4 Relationship between measurement errors and image coverage metrics

**Figure 10:**
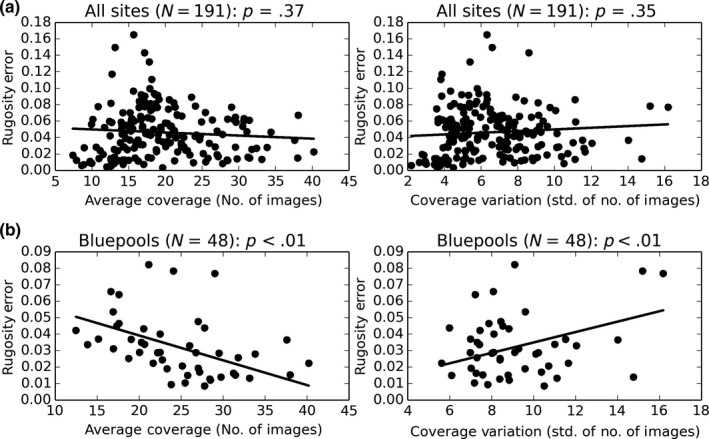
2 × 2 m quadrat rugosity errors (variation) versus coverage variation during image collection (number of images covering quadrat (average coverage) and standard deviation of number of images covering any part of the quadrat (coverage variation), per survey) for all sites (a) and using data selected from the Blue pools site only (b)

Correlations between rugosity measurement errors and image coverage metrics (average number of covering images and variation in covering images) were weak and found to be nonsignificant using OLS when using quadrat data from all sites (Figure 10a, Table 4a). Analyses were reperformed using data for each site one at a time (four separate models, one for each site); only the Blue pools site model exhibited a statistically significant correlation between rugosity measurement error and coverage parameters (Figure 10b, Table 4b). Errors had a slight negative correlation to the average coverage (number of images) and slight positive correlation to the coverage variation (standard deviation in number of images touching any part of the quadrat), although both relationships were weak (low coefficient values).

**Table 4:** Results for ordinary least squares (OLS) regression of quadrat rugosity error (response variable) predicted by average coverage and coverage variation (std) (σ_SR_ ~ avg. cov. + cov. var.). (a) Data from all sites. (b) Data from Blue pools site only

Predictor	Coefficient	Std. error	*p*‐value
(a) All sites: number of observations = 191, residual degrees of freedom = 188, Adj. *R* ^2^ = .006			
Intercept	0.0470	0.007	*p* < .01
Average coverage	−0.0004	<0.001	*p* = 0.369
Coverage variation	0.0010	0.001	*p* = .352
(b) Blue pools: number of observations = 48, residual degrees of freedom = 45, Adj. *R* ^2^ = .306			
Intercept	0.0404	0.011	*p* < .01
Average coverage	−0.0015	<0.001	*p* < .01
Coverage variation	0.0032	0.001	*p* < .01

### 3.5 Relationship between measurement errors, surface rugosity, and dominant morphotype

**Figure 11:**
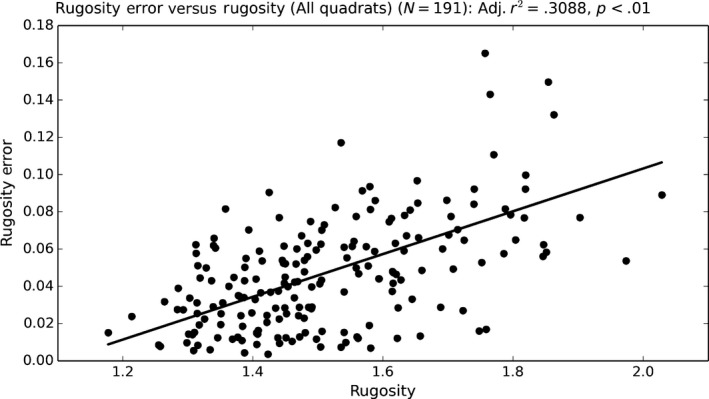
Relationships between 2 × 2 m quadrat rugosity errors and quadrat rugosity modeled using OLS

**Figure 12:**
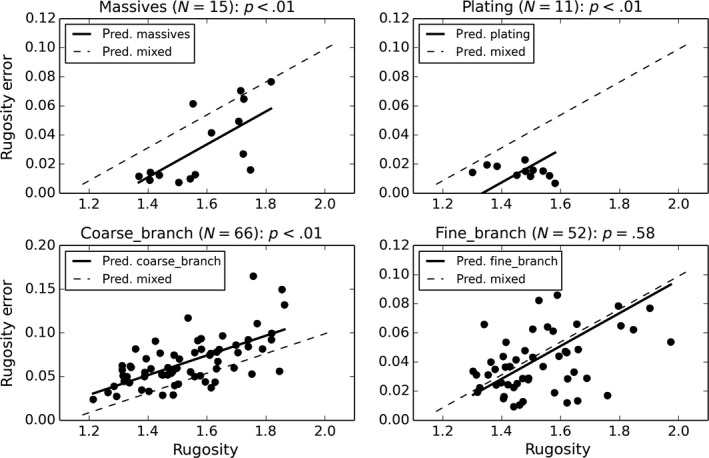
Relationships between 2 × 2 m quadrat rugosity errors and quadrat rugosity categorized by dominant coral morphotype coverage class modeled using OLS (adj. *R* ^2^ = .473). Model predicted rugosity error versus rugosity is shown for each class (solid line) and compared to the model predicted relationship for “mixed” type quadrats (dashed line)

Rugosity measurement errors exhibited statistically significant positive correlations to actual rugosity (Figure 11, Table 5a, adjusted *R* ^2^ = .309). When dominant coral morphotype within each quadrat was added to the model as a categorical variable, model fit improved from *R* ^2^ = .309 to *R* ^2^ = .473 and three of the four coral morphotypes had statistically significant model coefficients (Table 5b), when compared to the “Mixed” quadrat type base category. Quadrats dominated by “Massives” and “Plating” morphotypes had lower mean rugosity errors per quadrat rugosity, whereas “Coarse Branching” morphotypes had higher mean rugosity errors per quadrat rugosity when compared to “Mixed” quadrats (Figure 12). Quadrats dominated by “Fine‐Branching” morphotypes had no significant deviation in rugosity error from “Mixed” quadrats per quadrat rugosity. Coarse branching *Acropora* morphotypes likely exhibited higher errors owing to the way in which branches occlude each other, making surface reconstruction using a limited number of images (that cannot see through the branches) difficult as feature correspondences between images are more difficult to find. Massives and plating morphotypes likely exhibited smaller errors as the slowly undulating surfaces of these coral morphs are more easily observable from the multiple camera perspectives collected.

**Table 5:** Results for ordinary least squares (OLS) regression of quadrat rugosity error (response variable) predicted by (a) rugosity only and (b) rugosity and quadrat‐dominant coral morphotype

Predictor	Coefficient	Std. error	*p*‐value
(a) Model 1 (σ_SR_ ~ SR): number of observations = 191, residual degrees of freedom = 189, Adj. *R* ^2^ = .309			
Intercept	−0.1264	0.019	*p* < .01
Rugosity	0.1148	0.012	*p* < .01
(b) Model 2 (σ_SR_ ~ SR + C(type)): number of observations = 191, residual degrees of freedom = 185, Adj. *R* ^2^ = .473			
Intercept	−0.1323	0.017	*p* < .01
Rugosity	0.1130	0.011	*p* < .01
Type = massives	−0.0202	0.007	*p* < .01
Type = plating	−0.0238	0.008	*p* < .01
Type = coarse_branch	0.0204	0.005	*p* < .01
Type = fine_branch	−0.0028	0.005	*p* = .582

## 4 DISCUSSION

Our study has provided for the first time a detailed quantification of errors in structural complexity measurements made using photogrammetry/structure‐from‐motion and factors inducing errors. In‐water SR measurements consistently underestimated SR when compared to in‐air SR values, but were highly repeatable with a 4.5% precision SR standard error (Question 1). We observed significant per‐survey biases in SR measurements of up to 7.5% of the variation in measurements across a site. This indicates that variations in environmental conditions and/or survey technique employed at a single point in time had a large influence on the final SR measurement made (Question 2). SR measurement errors varied significantly with image density or image coverage pattern at one site of the four sites considered (Question 3a). Our results indicated that SR measurement errors exhibited a significant relationship with the underlying SR and dominant coral morphotype present (Question 3‐b,c). We were able to quantify the positive relationship between complexity measurement errors and the actual complexity itself, and further show that per‐unit complexity, some coral morphotypes exhibited above‐average errors (i.e., branching morphs) and others exhibited below‐average errors (i.e., massives and plating morphs). Our results have important implications for measuring actual habitat complexity change when changes are subtle, and highlight the importance on standardizing survey and postprocessing procedures as much as is possible, while targeting measurements to times when environmental conditions are similar among sites.

To the best of our knowledge, this is the first test of the accuracy of in‐water photogrammetric SR measurements for a medium size area (>1 m^2^). Previous studies that have tested the accuracy of photogrammetric SR measurements at the coral colony scale (cm^2^) (Figueira et al., 2015) have shown photogrammetric estimates of SR to underestimate actual SR, but with high precision. Our results have potential implications for studies utilizing measurements of complexity from various sources (e.g., combinations of measurements using structure‐from‐motion/3D modeling and traditional methods such as chain‐and‐tape) and indicate that calibration or correction factors between the two measurements types must be established before measurements can be compared. In future work, it will be important to determine whether the offsets observed in this study vary with different surface complexities, through imagery of artificial reefs varying in surface features.

The lack of a clear pattern in the errors across single versus multiple days meant we were unable to disambiguate between the relative magnitude of errors due to changing environmental conditions versus deviations in operator controlled survey trajectory or postprocessing routines. We observed that increasing image coverage resulted in lower complexity measurement error, likely due to more views being available to reconstruct parts of the observed surface that are partially occluded or not easily observable from all views. Higher variation in image coverage (some surfaces receiving many more or less views than others) resulted in less precise complexity measurements, likely due to inconsistencies between surfaces reconstructed from high densities versus low densities of image features. The strength of these relationships was, however, weak, probably due to the high base level of image coverage in the survey techniques employed. Typical structure‐from‐motion and associated surface reconstruction techniques require only three to five observations of each spatial point (Johnson‐Roberson et al., 2010), whereas our survey techniques encompassed 5–40 views. We have encountered difficulties in the postprocessing of 3D surfaces due to lack of image coverage, indicating that image coverage is an important factor in generating accurate 3D surface information and hence SR measurements (Bryson, per SOBS). Thus, the relationships between measurement error and coverage might not be as straightforward as the linear models considered here.

The advantages of using photogrammetric techniques for measuring structural attributes of coral reefs mean that these techniques are likely to be employed more widely within ecologically focused research and monitoring programs in the future (Burns et al., 2016; Ferrari, McKinnon, et al., 2016; Leon et al., 2015). In the context of monitoring, the quantitative models generated by this study provide a basis for predicting SR measurement error based on the level of complexity measured and knowledge of the coral morphotype imaged. Knowledge of measurement error and precision is an important factor to determine whether changes in an environment following a disturbance event are significant or not, where the statistical power to detect changes depends on the number of samples and the sample variance (Cohen, 1988; Green & Smith, 1997). Our results provide quantitative estimates of sample variance (depending on dominant coral morphologies) that could be used to determine the appropriate number of samples (quadrats) necessary for a given level of statistical power of experimental designs intended to detect changes in complexity. The optimization of sampling effort with appropriate consideration to statistical power is particularly important in the context of monitoring programs with limited resources (Hill & Wilkinson, 2004). Studies similar to ours have examined the variance associated with different measurement techniques for coral coverage (Jokiel et al., 2015; Leujak & Ormond, 2007), coral bleaching (Josephitis, Wilson, Moore, & Field, 2012), and benthic composition and visual complexity (Pante & Dunstan, 2012; Wilson et al., 2007).

The survey techniques presented here are designed to observe habitats from above, limiting the ability to resolve space underneath large overhanging structures, such as under large plating coral morphotypes. Goatley and Bellwood (2011) discuss the issues of horizontal planar views and sampling of coral reefs as missing information on the 3D structure of coral habitats, where the structural complexity and cover of organisms below the dominating upper “canopy” is missed. The photogrammetric techniques presented here can be adapted to measuring 3D structure on the underside of corals by adding photographic views of these points (e.g., Bythell et al., 2001; Courtney et al., 2007) but with increased cost of image acquisition and sampling effort per sampling unit. The results presented here are more likely to represent the realities of sampling using imagery in real monitoring programs in which the use of planar views maximizes the effective sampling area observable for a given constraint in sampling effort (Hill & Wilkinson, 2004).

## CONFLICT OF INTEREST

None declared.
